# Genetic Alterations in Medullary Thyroid Cancer: Diagnostic and Prognostic Markers

**DOI:** 10.2174/138920211798120835

**Published:** 2011-12

**Authors:** Taccaliti A, Silvetti F, Palmonella G, Boscaro M

**Affiliations:** Division of Endocrinology, University Polytechnic of Marche, Ancona, Italy

**Keywords:** Medullary Thyroid Carcinoma, RET proto-oncogene, diagnostic genetic markers, prognostic genetic markers.

## Abstract

Medullary thyroid carcinoma (MTC) is a rare calcitonin producing neuroendocrine tumour that originates from the parafollicular C-cells of the thyroid gland. The RET proto-oncogene encodes the RET receptor tyrosine kinase, with consequently essential roles in cell survival, differentiation and proliferation. Somatic or germline mutations of the RET gene play an important role in this neoplasm in development of sporadic and familial forms, respectively. Genetic diagnosis has an important role in differentiating sporadic from familiar MTC. Furthermore, depending on the location of the mutation, patients can be classified into risk classes. Therefore, genetic screening of the RET gene plays a critical role not only in diagnosis but also in assessing the prognosis and course of MTC.

## INTRODUCTION

Medullary Thyroid Carcinoma (MTC) is a rare neuroendocrine tumour arising from the calcitonin (Ct) producing parafollicular C cells of the thyroid. Serum Ct levels are elevated in C cell diseases, making it a sensitive biochemical marker for MTC. Recently a systematic review of autopsies has demonstrated that a small number of people in the general population have an occult MTC with a prevalence of 0.14% reaching to 0.42% when associated to Ct staining. MTC size was less than one centimetre, occurring prevalently in patients older than 60 years, with no gender difference [1].

MTC accounts for approximately 5-8% of all thyroid cancer. Clinically, MTC is mainly sporadic (70-80%), but hereditary pattern is present in 20-30% of cases, transmitted as an autosomal dominant trait [2]. 

Sporadic form of MTC is observed in 60-70 years old patients with a palpable thyroid nodule indistinguishable from any other thyroid nodule. Neck lymph node metastases are detected in at least 50% of patients and may reveal the disease. Metastases outside the neck, in liver, lungs or bones, are initially present in 10 - 20% of the cases.

Hereditary MTC may be part of “multiple endocrine neoplasia type 2” (MEN2) and is divided into three clinical forms:

- MEN2a is characterised by the presence of MTC in combination with pheochromocytoma and/or hyperpathy-roidism. Cutaneous lichen amyloidosis has been observed in some families [3] and Hirschsprung’s disease observed in a few families with MEN2a [4]. The typical onset of this condition is in the third or fourth decade of life. Nearly the 100% of gene carriers will develop MTC, but this depends on the mutation [5]. The risk of developing unilateral or bilateral pheochromocytoma is as high as 57% (both for germline mutations of codon 918 and 634), and 15– 30% of codon 634 mutations carriers will develop hyperpathyroidism [5,6].
- MEN2b in which MTC is accompanied by pheochromocytoma, multiple mucosal neuromas and marfanoid habitus. MEN2b is the most rare and aggressive form of MEN 2 based on its development of MTC earlier in life, usually before the age of 5-10 years. It is frequently associated with extension beyond the thyroid capsule, with lymph node and distant metastasis at the time of diagnosis [7]. Patients also have chronic constipation and colonic cramping due to the presence of megacolon disorder. More than 50% of cases are “*de novo”* germline mutations [8]. The higher mortality rate of MEN 2B reflects its more advanced stage at presentation, rather than the tumour behaviour once established [9-11].
- FMTC (Familiar Medullary Thyroid Carcinoma) occurs when MTC is the only clinical feature. Clinical presentation of cancer is at a later age and a relatively more favourable prognosis. The most rigid definition is multigenerational transmission of MTC in which no family member has pheochromocytoma or hyperpathyroidism; a less rigid definition is the presence of MTC in four affected family members without other manifestations of MEN 2A [12].

Histology is peculiar in hereditary MTC; C-cells hyperplasia is always associated with hereditary MTC with bilaterality and multicentricity as a consequence when the patients over 5-years old, carry the codon 918 or codon 634 mutations. Whereas, sporadic MTC generally presents as a single tumour confined to one thyroid lobe, except for a 5-9% of patients [13]. Tumour metastasizes early to paratracheal and lateral cervical lymph nodes; lymph nodes metastasis are found in 20-30% of patients with MTC < 1 cm in diameter, in 50% with tumour > 2 < 4 cm and in up 90% of the patients with more than 4 cm in diameter or infiltrating surround thyroid tissues. The prognosis of MTC is intermedia between well differentiate thyroid carcinomas and anaplastic thyroid cancer. It is worse respect to papillary and follicular thyroid cancer and better to anaplastic thyroid cancer. Therefore, an early diagnosis is fundamental for a good prognosis in these patients [14-16].

Genetic abnormalities are present in MTC, and hereditary forms are characterised by germline mutations while sporadic MTC showed somatic alterations in 40-60% of patients.

## RET PROTONCOGENE

The “REarrangement during Transfection” (RET) proto-oncogene, which is primarily expressed in neuronal crest-derived and urogenital progenitor cells, is required for the development of enteric nervous system, kidney morphogenesis and differentiation of spermatogonia [17].

The predisposing gene for hereditary MTC was RET proto-oncogene localized to centromeric chromosome 10 by linkage analysis in 1987, and germline mutations of the RET proto-oncogene were identified in 1993 in MEN2A, MEN2B and FMTC [18-20]. 

The human RET gene maps on 10q11.2 and is composed of 21 exons with a size of about 55 kb [21, 22]. The RET proto-oncogene encodes a tyrosin kinase transmembrane receptor which is mainly expressed in neural crest-derived cell lineages as parafollicular C cells, adrenal medullary cells and parathyroid cells. RET receptor plays a pivotal role in regulating cell proliferation, migration, and differentiation. The plasma membrane tyrosine kinase receptor is characterised by three different domains: i) the extracellular domain, ii) the transmembrane domain, and iii) the intracellular tyrosine kinase domain. The extracellular domain contains four Ca2-dependent cell adhesion (cadherin)-like domains that are thought to induce and stabilize conformational changes for interaction with ligands and co-receptors, and a cysteine-rich region responsible for the tertiary structure and ligand-induced dimerization of two RET molecules formation of dimers [23, 24]. The transmembrane domain ensures the close proximity of the RET proto-oncogene monomers through non-covalent receptor-receptor interactions [25]. The intracellular region comprises a juxta-membrane domain, and two tyrosine kinase subdomains (TK1 and TK2) that are involved in the activation of numerous intracellular signal transduction pathways.

**Fig. (1):**
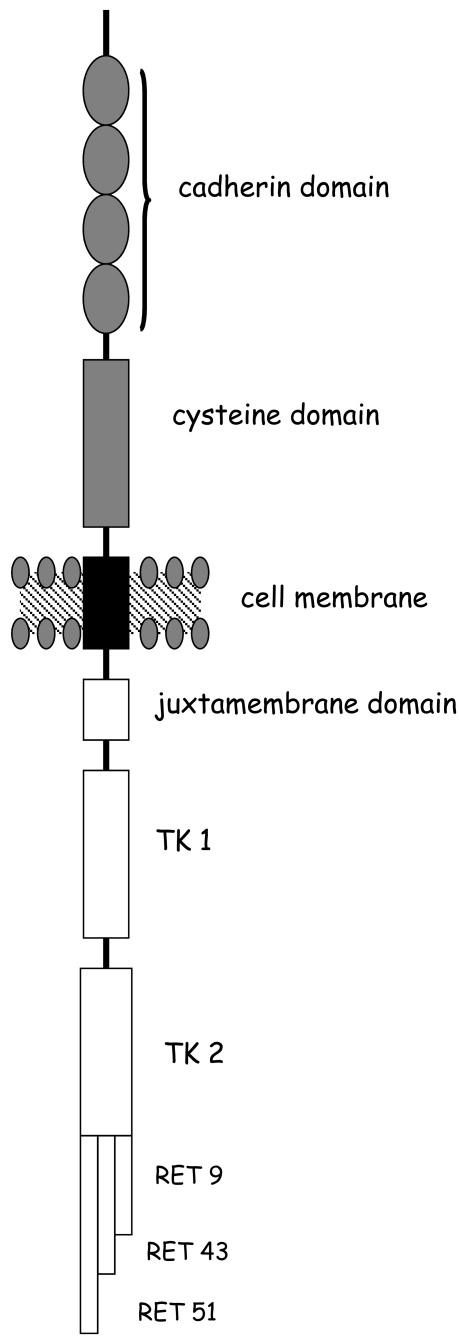
Shows the RET receptor consists of 3 parts. Grey extracellular cadherin-like region and the region rich in cysteine. Black transmembrane region and Red intracytoplasmic region with tyrosine kinase activity. They are also representing the three isoforms (RET9, RET43 and RET51).

Alternate splicing of RET produces three isoforms with either 9, 43, or 51 amino acids at the C terminus, referred to as RET9, RET43, or RET51 (Fig. **1**).

Under normal conditions RET can be activated by a complex of coreceptors and ligands. These belong to two groups of proteins: the GDNF (Glial cell line-Derived Neurotrophic Factor) and the glycosylphosphatidylinositol-anchored GDNF- family α receptor. Binding of the ligand to the extracellular domain of the RET tyrosin kinase induces receptor dimerization and autophosphorylation, creating intracellular binding sites for signalling proteins with the subsequent activation of multiple signalling pathways. In fact, autophosphorylation at specific cytoplasmatic Tyr residues provides the docking site for several adaptor/signalling proteins and is a requirement for the receptor signal transduction.

Alterations of the RET proto-oncogene are involved in the development of different human diseases. “Gain of function” mutations, leading to aberrant activation of RET, are specific oncogenic events in the thyroid gland. RET oncoproteins expressed in the thyroid carcinomas are characterised by constitutive ligand-independent tyrosine kinase activity necessary for their transformation ability [26].

## RET ACTIVATION IN MTC

Mutated RET plays a very significant role in the development of human neuroendocrine tumours and tumour syndromes. The activation of oncogenic RET depends on the location of the amino acid change. Normally, cysteine residues are involved in intramolecular disulfide bonds in wild-type RET. Mutations in the extracellular cysteine-rich domain convert a cysteine residue into a noncysteine residue. These mutations leave an unpaired cysteine residue in a RET monomer to form an aberrant intermolecular disulfide bond with another mutated monomer. The two mutated RET molecules are constitutively dimerized and activated. Mutations in the intracellular tyrosine kinase domain activate tyrosines in the kinase domain and alter its substrate specificity due to structural changes of the binding pocket of the tyrosine kinase domain. They lead to aberrant phosphorylation of substrates preferred by cytoplasmic tyrosine kinases [27]. Consequently, the mutated RET no longer needs dimerization to become active [28].

MTCs are characterised by activating mutations of RET proto-oncogene which occur prevalently as point alteration of codons. In 98% of MEN 2a families, a germ-line activating RET mutation can be detected [5]. Germline RET mutations indicate hereditary MTC and determine the lifetime risk for developing MTC, which is nearly 100% for RET mutation carriers. A high prevalence of “*de novo”* RET mutations (over 50%) has been identified in MEN 2b patients, and to a lesser extent in MEN 2a⁄FMTC patients. Also, germ-line RET mutations are frequently detected in apparently sporadic MTC patients, indicating the importance of genetic testing in all MTC patients, even without a clear indication for hereditary disease.

Somatic RET mutations can be detected in tumour tissue of 40-60% of sporadic MTC patients. The contribution to tumour development of somatic RET mutations in MTC pathogenesis is unclear. Somatic RET mutations are not consistently distributed within primary tumours and metastases, indicating that the mutation can occur during progression of the tumour or that MTC is a disease of polyclonal origin [29]. Probably in these cases, somatic RET mutations merely contribute to the disease phenotype instead of causing it.

Fig. (**2**) shows the most frequent germlines of somatic mutations.

## GERMLINE MUTATIONS

In 98% of MEN2a patients, mutations occur in one of the five cysteines in the extracellular cysteine-rich domain of RET and are localized in exons 10-11 of RET oncogene. The majority of MEN2b patients, about 95% of cases, carry the mutations in the intracellular tyrosin-kinase domain of RET localized in exon 16. In FMTC, mutations affect either extracellular cystein or intracellular domain of RET concerning all previous exons [30-32].

Extracellular cysteine RET mutations exert constitutive kinase activity consequence which is independent from ligand. In the cases of mutation M918T, constitutive RET activation probably results from disruption of an auto-inhibited head-to-tail RET tyrosine kinase homodimer [33].

Some studies have found an overlap between RET mutations and clinical subtypes of MEN2, identifying the genotype-phenotype very substantial [5, 30, 34]*.* In particular, hyperparathyroidism in MEN2a is associated with high frequency, with the mutation 634, especially in the Cys-Arg substitution, less frequently with mutations in codons 609, 611, 618, 620, 790, 791. Moreover, the mutation of codon 918 in exon 16 is associated with the MEN2b present in 95% of cases and is usually a “*de novo”* mutation that appeared for the first time in sick patients and not inherited by their healthy parents.

In both ItaMEN [35] and the German series [36], the majority of RET families harboured codon 634 mutations (37% of Italian *vs*. 41% of German RET families). Codon 804 and 891 mutations affected some 22% and 10% of Italian *vs*. 6% and 2% of German RET families. Conversely, codon 790 and 791 mutations were more common in German than Italian RET families (12% *vs*. 3% and 7% *vs*. <1%).

Moreover, a high prevalence of several mutations affecting non-cysteine codons, some of which were never reported before as we demonstrated in a family of FMTC with a mutation of Cys 515 in exon 8 which induced gain of function of RET gene [37]. The different ethnic origins of the patients and the particular attention given to analysing apparently sporadic MTC for RET germlines mutations may explain these Italian data.

The correlation between genotype and phenotype is not only for the clinical but also the age of onset of MTC and its aggressiveness. Guidelines in 2001 have suggested to consider the age of surgery according to three risk categories according to RET mutation (levels 1, 2, 3) [5]. The 2009 American Thyroid Association (ATA) guidelines established a four-level risk categorization (classes A, B, C, D) recognizing the most aggressive course of the 634 mutation within level 2 [38]. For patients with mutation at codon 918 (MEN 2b), surgery is recommended during the first year of life, and as soon as possible (level 3 in the past, D currently). Regarding mutations in exons 10 and 11, prophylactic surgery is proposed before the age of 5 (level 2, ATA class C). Finally, for mutations in exons 13, 14, and 15, prophylactic surgery may be delayed beyond the age of 5, in the absence of any evidence of C cell disease (level 1, A) [4, 37]. However, these recommendations were mainly based on the age-related tumour, node metastasis (TNM) status rather than long-term follow-up data. Surgery should include a total thyroidectomy, but timing and extent of lymph node dissection are still a matter of debate, being currently based on the germline mutation, the age at surgery, and on any evidence of disease, including neck ultrasonography and serum calcitonin (CT) levels [38-41].

In a German Austrian study authors have demonstrated that patients with rare mutations at codons 790, 791 and 804 showed differ onset and outcome. In particular, there is a significant difference in MTC development with less extensive C-cell disease, higher cure rate and more frequent additional endocrinopathies in carriers of *RET *codon 791 mutations compared with carriers of codons 790 and 804 mutations [42]*.*

Recently a French study has demonstrated that the prognostic factor of disease free survival after surgery in young patients with RET germline mutation is best predicted by TNM staging and preoperative basal CT level below 30 pg/ml. Basal CT, class D genotype, and age constitute key determinants to decide preoperatively timely surgery [43].

Moreover, genetic screening of germline RET mutations permits to identify unsuspected FMTC in apparently sporadic MTC patients. Therefore, RET genetic screening of patients with apparently sporadic MTC represents a major tool for the preclinical diagnosis and early treatment of unsuspected affected family members and allows the identification of a relevant percentage of hidden FMTC.

Given the high chance of a RET gene carrier developing MTC at some point during their life, these patients should be offered prophylactic thyroidectomy. In cases of MEN 2a/FMTC mutations of ATA level C risk, prophylactic total thyroidectomy should be carried out before 5 years of age [38]. In patients with RET mutations of ATA level A and B risk, prophylactic thyroidectomy may be delayed beyond the first 5 years in the setting of a normal basal and/or stimulated serum CT and normal annual cervical ultrasound starting at 5 years of age [38]. Prophylactic level VI central compartment neck dissection may not be necessary in patients with MEN 2a/FMTC who undergo prophylactic thyroidectomy within their first 3-5 years of life unless there is clinical or radiological evidence of lymph node metastases, thyroid nodules >5 mm in size or a serum basal CT >40 pg/ml in a child >6 months old [38]. Children with MEN 2b (ATA level D risk) should have thyroidectomy as soon as possible, preferably within the first year of life. Prophylactic level VI neck dissection may not be necessary in patients with MEN 2b, unless there is clinical or radiological evidence of lymph node metastases [38]. In RET-mutation-positive patients, screening for pheochromocytoma, including annual plasma metanephrines and normetanephrines, or 24-h urine collection for metanephrines and normetanephrines, begins by 8 years of age in carriers of the RET mutation associated with MEN 2b and codons 630 and 634, and by 20 years of age in carriers of other MEN 2a RET mutations. Patients with RET mutation associated with FMTC alone should be screened periodically from 20 years of age. Abdominal imaging is not indicated in the absence of symptoms or biochemical data [38]. Screening for hyperparathyroidism should be carried out with the same interval by measuring serum calcium and parathyroid hormone.

## SOMATIC MUTATIONS

**Fig. (2):**
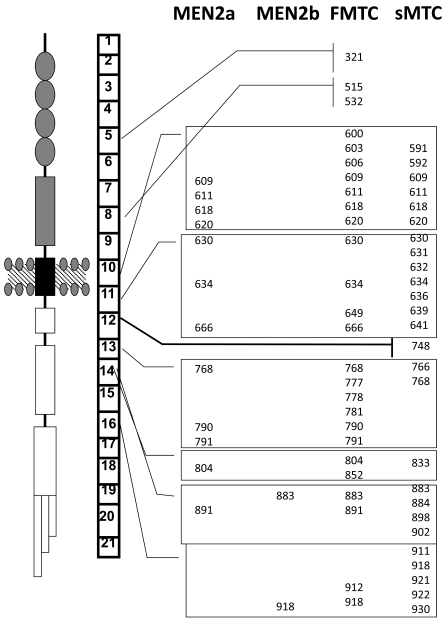
Shows the RET receptor, the gene with its exons and the most common mutations in hereditary and sporadic MTC.

Somatic mutations are demonstrated in 40 to 60% of sporadic MTCs. They occur in the tyrosine kinase domain and in cysteine rich domain of RET gene. Mutations located in the intracellular region represent approximately one-third of all sporadic MTC and only a few mutations or deletion have been reported in the extracellular cystein-rich domain [44-46]. The most common mutation is M918T, although many other codons could show mutations (Fig. **2**) [29, 47-49]. 

In few cases of sporadic MTC a deletion of codons Glu632 and Leu633 was identified. These mutations activate more effectively the RET gene in comparison to the Cys 634Arg missense mutation and they induce stable dimers formation in the absence of ligand [50].

More aggressive sporadic MTC showed the M918T RET mutation in about 30-50% [51]. It has been demonstrated that the presence of a somatic RET mutation (M918T) correlates with stage of the disease, a high probability to have persistence of the disease after total thyroidectomy, increased chance of recurrence and metastatic potential, and a reduced survival. This indicates that the presence of somatic RET mutations may function as a prognostic biomarker [52].

Moreover, correlation was identified between the presence of somatic mutations with more advanced pathological TNM stage [53].

## RET PROTO-ONCOGENE POLYMORPHISMS

Single nucleotide polymorphisms (SNPs) are allelic variants of a gene that occur when one nucleotide is replaced without alteration of functional activity of the encoded protein. SNPs have been reported to function as genetic modifiers of RET protooncogene mutations, resulting in the expression of MTC and PTC. Specifically, SNP G691S in exon 11 and SNP S904S in exon 15 are believed to be responsible for the development of MTC in patients with germline RET proto-oncogene mutations. It also has been reported that SNPs G691S and S904S appear to influence the age of onset of MTC in patients with MEN 2A syndrome. Several reports have shown that patients homozygous for these polymorphisms were, on average, diagnosed with MTC 10 years earlier than patients with MEN 2A bearing heterozygous or wild-type haplotype [54]. Siqueira and coworkers recently demonstrated higher frequencies of RET polymorphism (L769L, S836S, S904S, G691S) in hereditary and sporadic MTC (102 and 81 patients respectively) than controls. They also detected that individuals harbouring the S836S variant were younger and showed a higher percentage of lymph node and distant metastases [55]. Sromek *et al*. compared the frequency of three polymorphic changes in the RET proto-oncogene (L769L, S836S and S904S) between patients with MTC and the general population. In their paper they evidenced the predisposition role of SNP L769L in the developing of sporadic MTC and also in the lowering the age of onset of MTC in carriers of the homozygous SNP L769L. [56]. At least, Pasquali and his colleagues recently reported a new SNP, V109G. They detected that, in sporadic MTC, the V109G polymorphism correlates with a more favourable disease progression than the wild-type allele [57].

## RAS MUTATIONS IN MTC

Mutations of RAS occur with variably frequency in all types of thyroid follicular cell-derived tumours (papillary and follicular thyroid cancer) and discordant data have been published in MTC. The *RAS *genes (*H-RAS*, *K-RAS*, and *N-RAS*) encode highly related G-proteins that are located at the inner surface of the cell membrane and play a central role in the intracellular transduction of signals arising from cell membrane receptors tyrosine kinase and G-protein-coupled receptors. In its inactive state, RAS protein is bound to guanosine diphosphate (GDP). Upon activation, it releases GDP and binds guanosine triphosphate (GTP), activating the MAPK and other signalling pathway, such as PI3K/AKT. Normally, the activated RAS-GTP protein becomes quickly inactive due to its intrinsic guanosine triphosphatase (GTPase) activity and the action of cytoplasmic GTPase-activating proteins, which catalyse the conversion of the active GTP form to the inactive GDP-bound form. In many human neoplasms, point mutations occur in the discrete domains of the *RAS *gene, which result in either an increased affinity for GTP (mutations in codons 12 and 13) or inactivation of the autocatalytic GTPase function (mutations in codon 61). As a result, the mutant protein becomes permanently switched in the active position and constitutively actives its downstream signalling pathways [58]. Absence of H-RAS and K-RAS, as well N-RAS mutations in sporadic MTC was described in early reports except Okazaki *et al*. who identified a H-RAS mutation in one out of 10 sporadic MTC [59]. More recently, somatic mutations of H-RAS and K-RAS genes have been identified in sporadic MTC without RET mutation by Muora and coworkers and no mutations of N-RAS were detected. They hypothesise that activation of the protoncogenes RAS and RET is likely to represent alternative genetic events in MTC tumorigenesis [60]. Similar results were found by Goutas *et al*. They detected mutation of codon 12 of K-RAS in 18 (40.9%) out of 44 sporadic MTC [61].

## RET AS THERAPEUTIC TARGET IN MTC

Several small-molecule tyrosine kinase (TK) inhibitors directed towards RET kinase have been tested in preclinical and clinical studies and have been proven to be effective in the treatment of several neoplastic disease, including thyroid cancers [58]. After treatment with TK inhibitors in a large number of patients with thyroid cancers (25-81%) a stable disease state can be established, and some patients even show a partial response (2-33%). Verbeek and coworkers set out to compare the efficiency of four recently developed TK inhibitors, XL184, Vandetanib, Sunitinib and Axitinib, using three cell lines: the first derived from sporadic MTC expressing a C634W RET mutation, the second derived from metastatic sporadic MTC expressing a M918T RET mutation. They detected that all four TK inhibitors are capable of reducing cell proliferation, however, XL184 was found to be the most efficient inhibitor for MEN2A derived cells lines, whereas vandetanib proved to be the most potent inhibitor for MEN2B [62]. Recently has been evaluated the combination of a multikinase inhibitor (Sorafenib) with a farnesyltransferase inhibitor (Tipifarnib) in the treatment of thyroid tumours. Sorafenib is a potent oral multikinase inhibitor of Raf-1, platelet-derived growth factor receptor, RET, KIT, and vascular endothelial growth factor receptor-2. Tipifarnib is a selective oral farnesyltransferase inhibitor, inducing antiproliferative effects against various human tumour cell lines. The authors tested the hypothesis that more clinical activity should be achieved by using the combination of sorafenib and tipifarnib, given the importance of Ras/Raf/MAPK kinase/ERK and RET kinase in thyroid malignancies. At the end of the study, among patients with MTC, they evidenced a 38% of partial response and a 31% of stable disease at 6 months. Therefore, they have considered useful association of sorafenib and tipifarnib in the treatment of aggressive MTC [63].

## CONCLUSIONS

RET screening for detection of genetic mutations is essential for a correct approach in the management of MTC. All subjects with a diagnosis of MTC, even without a family history of MEN2 should be subjected to genetic analysis of RET, in order to confirm or exclude the inheritance of the disease. Indeed, as previously reported, a rate between 5-10% of sporadic forms had RET germline mutation. RET screening is also essential to assess first-degree relatives of proband to determine their possible carrier status for the relationship between type of mutation and the aggressiveness of disease, and the correct timing of prophylactic thyroidectomy. 

In sporadic form of MTC also the identification of somatic RET mutation has a prognostic value making it possible to select individuals who require a more careful follow-up. In fact, the presence of somatic mutations in specific codons of RET gene correlates with greater aggressiveness and worse prognosis. Therefore, the search for somatic mutations plays an important role in the clinical course and follow-up of patients with sporadic MTC.

Therefore, screening of the RET gene today is a milestone in the management of all patients with newly diagnosed MTC and all the family of a patient with known RET germline mutation.
